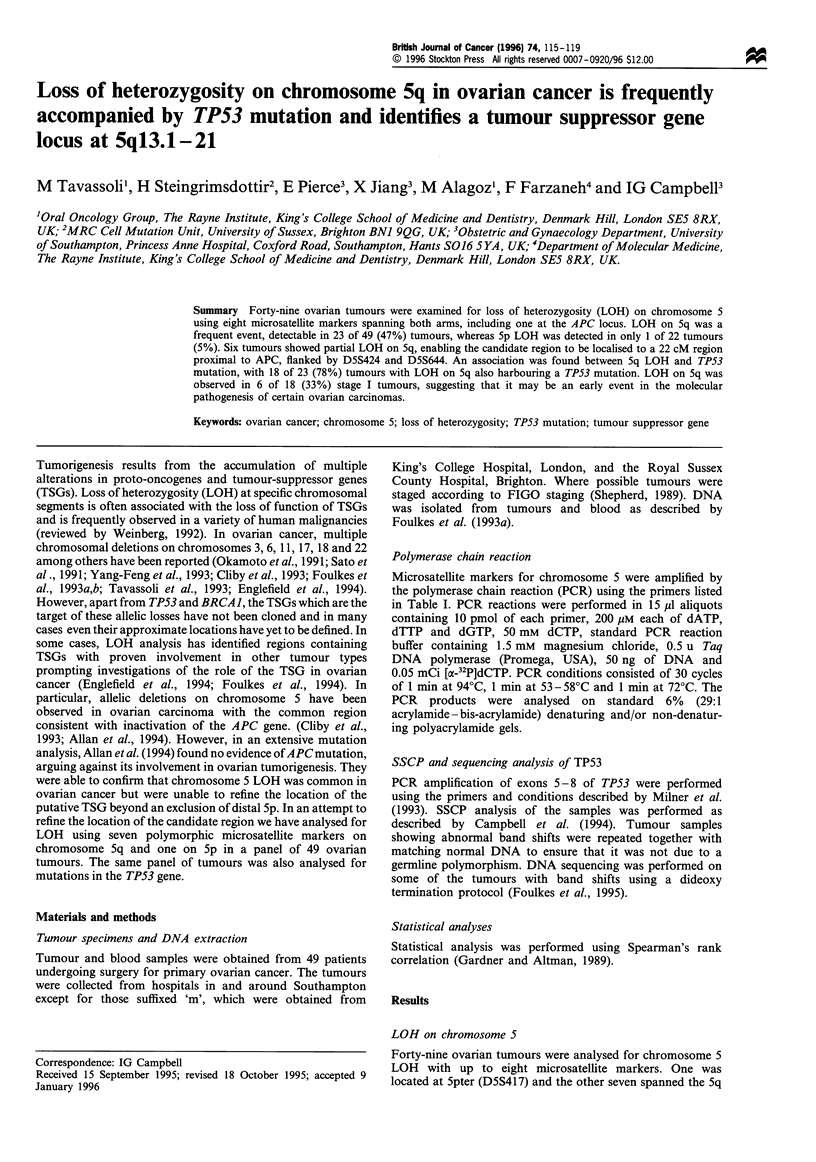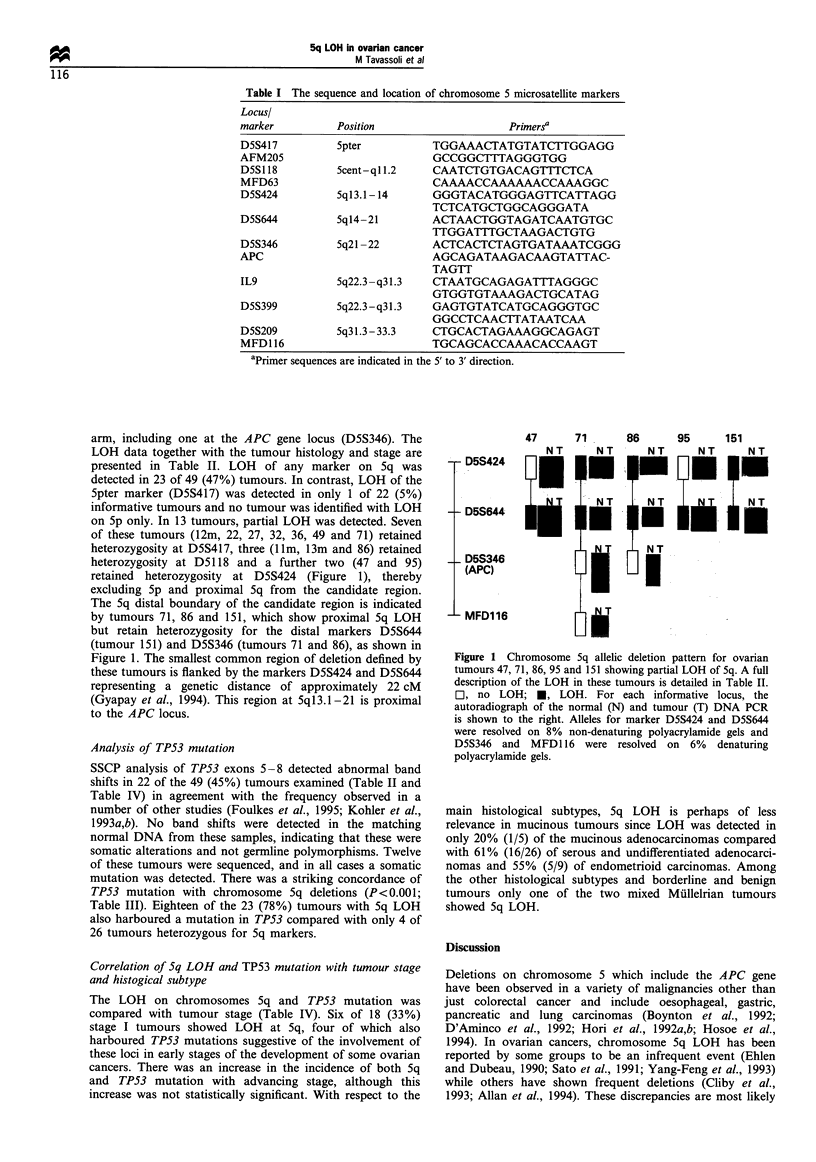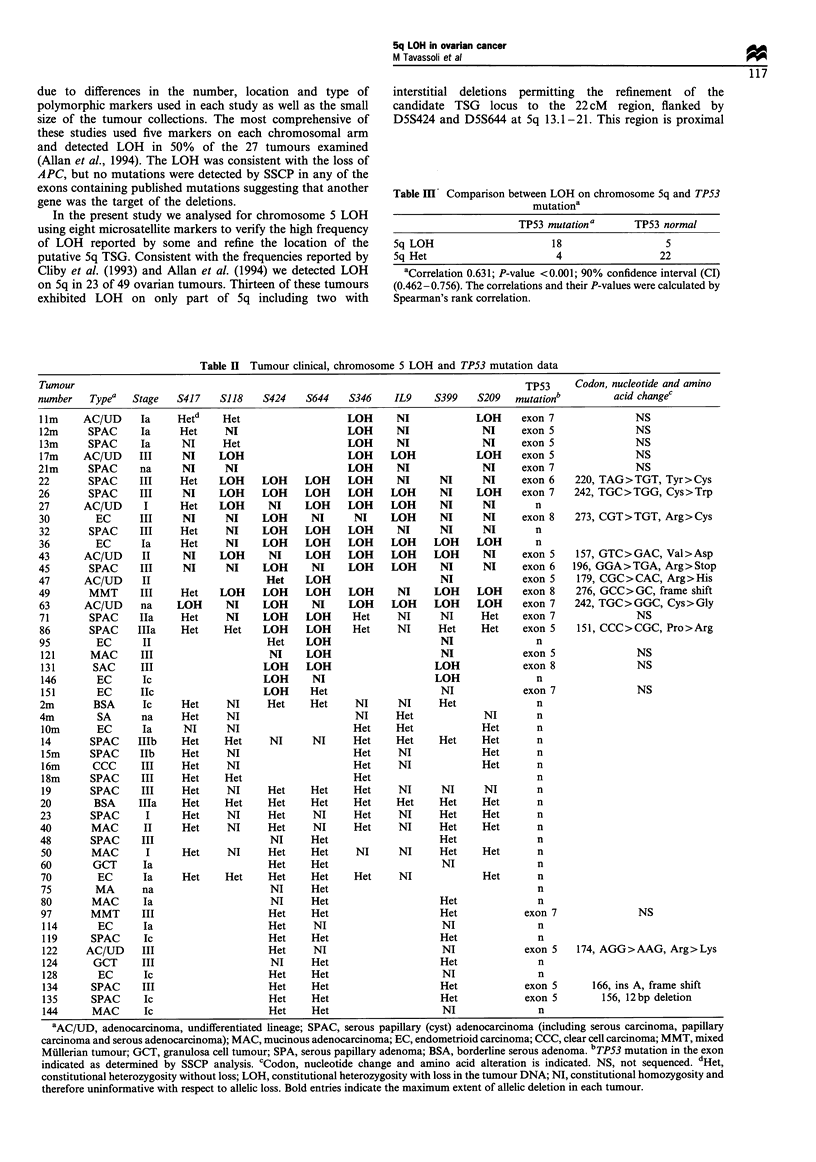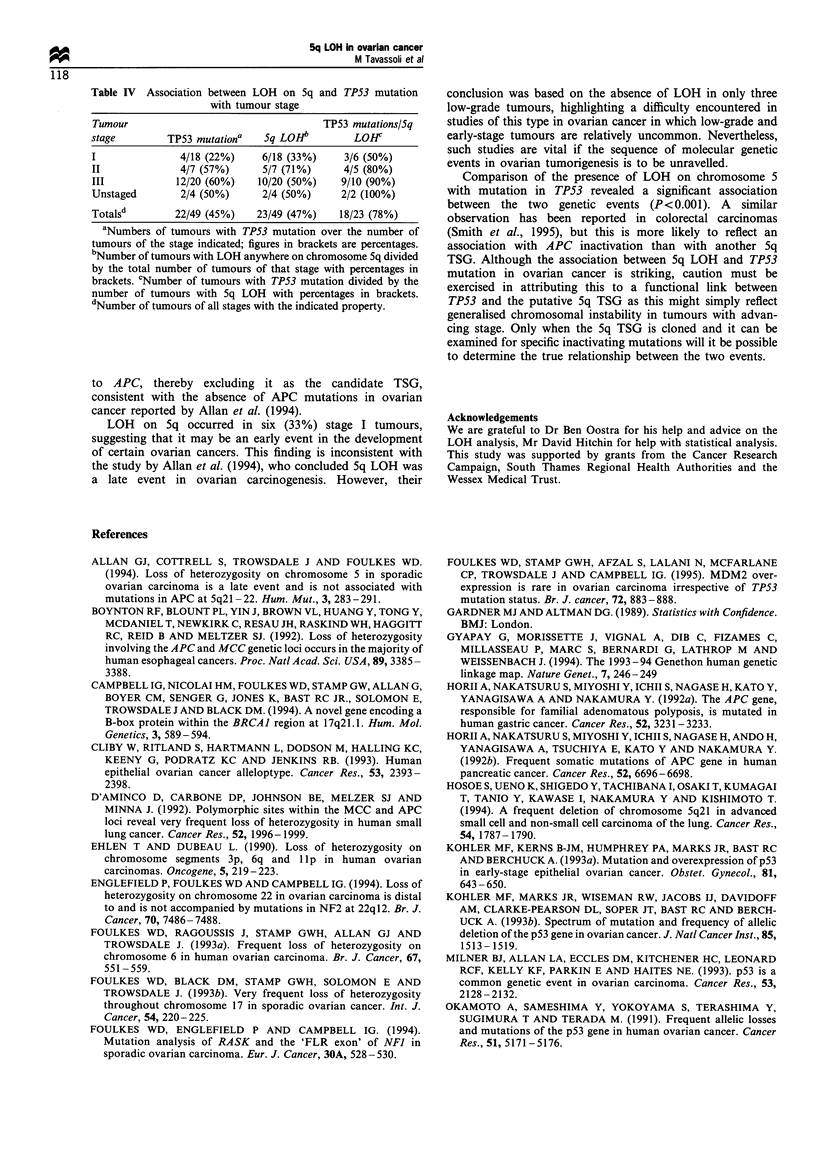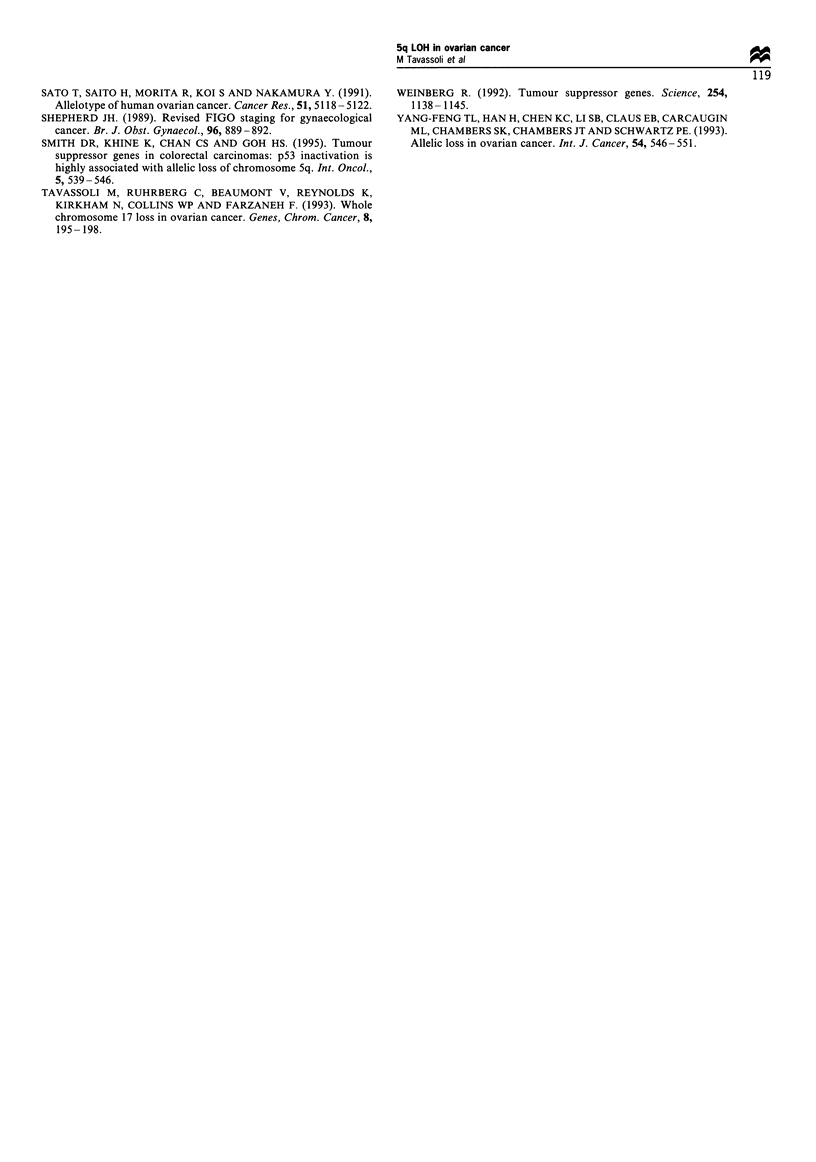# Loss of heterozygosity on chromosome 5q in ovarian cancer is frequently accompanied by TP53 mutation and identifies a tumour suppressor gene locus at 5q13.1-21.

**DOI:** 10.1038/bjc.1996.324

**Published:** 1996-07

**Authors:** M. Tavassoli, H. Steingrimsdottir, E. Pierce, X. Jiang, M. Alagoz, F. Farzaneh, I. G. Campbell

**Affiliations:** Rayne Institute, King's College School of Medicine and Dentistry, London, UK.

## Abstract

**Images:**


British Journal of Cancer (1996) 74, 115-119
© 1996 Stockton Press All rights reserved 0007-0920/96 $12.00

### Loss of heterozygosity on chromosome 5q in ovarian cancer is frequently accompanied by *TP53* mutation and identifies a tumour suppressor gene locus at 5q13.1-21

M Tavassoli[1], H Steingrimsdottir[2], E Pierce[3], X Jiang[3], M Alagoz[1], F Farzaneh[4] and IG Campbell[3]

*[1]Oral Oncology Group, The Rayne Institute, King's College School of Medicine and Dentistry, Denmark Hill, London SE5 8RX, UK; [2]MRC Cell Mutation Unit, University of Sussex, Brighton BN1 9QG, UK; [3]Obstetric and Gynaecology Department, University of Southampton, Princess Anne Hospital, Coxford Road, Southampton, Hants SO16 5YA, UK; [4]Department of Molecular Medicine, The Rayne Institute, King's College School of Medicine and Dentistry, Denmark Hill, London SE5 8RX, UK.*

**Summary** Forty-nine ovarian tumours were examined for loss of heterozygosity (LOH) on chromosome 5 using eight microsatellite markers spanning both arms, including one at the *APC* locus. LOH on 5q was a frequent event, detectable in 23 of 49 (47%) tumours, whereas 5p LOH was detected in only 1 of 22 tumours (5%). Six tumours showed partial LOH on 5q, enabling the candidate region to be localised to a 22 cM region proximal to APC, flanked by D5S424 and D5S644. An association was found between 5q LOH and *TP53* mutation, with 18 of 23 (78%) tumours with LOH on 5q also harbouring a *TP53* mutation. LOH on 5q was observed in 6 of 18 (33%) stage I tumours, suggesting that it may be an early event in the molecular pathogenesis of certain ovarian carcinomas.

**Keywords:** ovarian cancer; chromosome 5; loss of heterozygosity; *TP53* mutation; tumour suppressor gene

Tumorigenesis results from the accumulation of multiple alterations in proto-oncogenes and tumour-suppressor genes (TSGs). Loss of heterozygosity (LOH) at specific chromosomal segments is often associated with the loss of function of TSGs and is frequently observed in a variety of human malignancies (reviewed by Weinberg, 1992). In ovarian cancer, multiple chromosomal deletions on chromosomes 3, 6, 11, 17, 18 and 22 among others have been reported (Okamoto *et al*., 1991; Sato *et al*., 1991; Yang-Feng *et al*., 1993; Cliby *et al*., 1993; Foulkes *et al*., 1993*a*,*b*; Tavassoli *et al*., 1993; Englefield *et al*., 1994). However, apart from *TP53* and *BRCA1*, the TSGs which are the target of these allelic losses have not been cloned and in many cases even their approximate locations have yet to be defined. In some cases, LOH analysis has identified regions containing TSGs with proven involvement in other tumour types prompting investigations of the role of the TSG in ovarian cancer (Englefield *et al*., 1994; Foulkes *et al*., 1994). In particular, allelic deletions on chromosome 5 have been observed in ovarian carcinoma with the common region consistent with inactivation of the *APC* gene. (Cliby *et al*., 1993; Allan *et al*., 1994). However, in an extensive mutation analysis, Allan *et al*. (1994) found no evidence of *APC* mutation, arguing against its involvement in ovarian tumorigenesis. They were able to confirm that chromosome 5 LOH was common in ovarian cancer but were unable to refine the location of the putative TSG beyond an exclusion of distal 5p. In an attempt to refine the location of the candidate region we have analysed for LOH using seven polymorphic microsatellite markers on chromosome 5q and one on 5p in a panel of 49 ovarian tumours. The same panel of tumours was also analysed for mutations in the *TP53* gene.

### Materials and methods

### *Tumour specimens and DNA extraction*

Tumour and blood samples were obtained from 49 patients undergoing surgery for primary ovarian cancer. The tumours were collected from hospitals in and around Southampton except for those suffixed 'm', which were obtained from King's College Hospital, London, and the Royal Sussex County Hospital, Brighton. Where possible tumours were staged according to FIGO staging (Shepherd, 1989). DNA was isolated from tumours and blood as described by Foulkes *et al*. (1993*a*).

### *Polymerase chain reaction*

Microsatellite markers for chromosome 5 were amplified by the polymerase chain reaction (PCR) using the primers listed in Table I. PCR reactions were performed in 15 $\mu$l aliquots containing 10 pmol of each primer, 200 $\mu$M each of dATP, dTTP and dGTP, 50 mM dCTP, standard PCR reaction buffer containing 1.5 mM magnesium chloride, 0.5 u *Taq* DNA polymerase (Promega, USA), 50 ng of DNA and 0.05 mCi [$\alpha$-$^{32}$P]dCTP. PCR conditions consisted of 30 cycles of 1 min at 94°C, 1 min at 53-58°C and 1 min at 72°C. The PCR products were analysed on standard 6% (29:1 acrylamide-bis-acrylamide) denaturing and/or non-denaturing polyacrylamide gels.

### *SSCP and sequencing analysis of* TP53

PCR amplification of exons 5-8 of *TP53* were performed using the primers and conditions described by Milner *et al*. (1993). SSCP analysis of the samples was performed as described by Campbell *et al*. (1994). Tumour samples showing abnormal band shifts were repeated together with matching normal DNA to ensure that it was not due to a germline polymorphism. DNA sequencing was performed on some of the tumours with band shifts using a dideoxy termination protocol (Foulkes *et al*., 1995).

### *Statistical analyses*

Statistical analysis was performed using Spearman's rank correlation (Gardner and Altman, 1989).

### Results

### *LOH on chromosome 5*

Forty-nine ovarian tumours were analysed for chromosome 5 LOH with up to eight microsatellite markers. One was located at 5pter (D5S417) and the other seven spanned the 5q

Correspondence: IG Campbell
Received 15 September 1995; revised 18 October 1995; accepted 9 January 1996

**Table I** The sequence and location of chromosome 5 microsatellite markers

| *Locus/ marker* | *Position* | *Primers*[a] |
|---|---|---|
| D5S417 | 5pter | TGGAAACTATGTATCTTGGAGG |
| AFM205 | | GCCGGCTTTAGGGTGG |
| D5S118 | 5cent–q11.2 | CAATCTGTGACAGTTTCTCA |
| MFD63 | | CAAAACCAAAAAACCAAAGGC |
| D5S424 | 5q13.1–14 | GGGTACATGGGAGTTCATTAGG |
| | | TCTCATGCTGGCAGGGATA |
| D5S644 | 5q14–21 | ACTAACTGGTAGATCAATGTGC |
| | | TTGGATTTGCTAAGACTGTG |
| D5S346 | 5q21–22 | ACTCACTCTAGTGATAAATCGGG |
| APC | | AGCAGATAAGACAAGTATTAC-TAGTT |
| IL9 | 5q22.3–q31.3 | CTAATGCAGAGATTTAGGGC |
| | | GTGGTGTAAAGACTGCATAG |
| D5S399 | 5q22.3–q31.3 | GAGTGTATCATGCAGGGTGC |
| | | GGCCTCAACTTATAATCAA |
| D5S209 | 5q31.3–33.3 | CTGCACTAGAAAGGCAGAGT |
| MFD116 | | TGCAGCACCAAACACCAAGT |

[a]Primer sequences are indicated in the 5′ to 3′ direction.

arm, including one at the *APC* gene locus (D5S346). The LOH data together with the tumour histology and stage are presented in Table II. LOH of any marker on 5q was detected in 23 of 49 (47%) tumours. In contrast, LOH of the 5pter marker (D5S417) was detected in only 1 of 22 (5%) informative tumours and no tumour was identified with LOH on 5p only. In 13 tumours, partial LOH was detected. Seven of these tumours (12m, 22, 27, 32, 36, 49 and 71) retained heterozygosity at D5S417, three (11m, 13m and 86) retained heterozygosity at D5118 and a further two (47 and 95) retained heterozygosity at D5S424 (Figure 1), thereby excluding 5p and proximal 5q from the candidate region. The 5q distal boundary of the candidate region is indicated by tumours 71, 86 and 151, which show proximal 5q LOH but retain heterozygosity for the distal markers D5S644 (tumour 151) and D5S346 (tumours 71 and 86), as shown in Figure 1. The smallest common region of deletion defined by these tumours is flanked by the markers D5S424 and D5S644 representing a genetic distance of approximately 22 cM (Gyapay *et al*., 1994). This region at 5q13.1–21 is proximal to the *APC* locus.

*Analysis of TP53 mutation*

SSCP analysis of *TP53* exons 5–8 detected abnormal band shifts in 22 of the 49 (45%) tumours examined (Table II and Table IV) in agreement with the frequency observed in a number of other studies (Foulkes *et al*., 1995; Kohler *et al*., 1993*a,b*). No band shifts were detected in the matching normal DNA from these samples, indicating that these were somatic alterations and not germline polymorphisms. Twelve of these tumours were sequenced, and in all cases a somatic mutation was detected. There was a striking concordance of *TP53* mutation with chromosome 5q deletions ($P<0.001$; Table III). Eighteen of the 23 (78%) tumours with 5q LOH also harboured a mutation in *TP53* compared with only 4 of 26 tumours heterozygous for 5q markers.

*Correlation of 5q LOH and* TP53 *mutation with tumour stage and histogical subtype*

The LOH on chromosomes 5q and *TP53* mutation was compared with tumour stage (Table IV). Six of 18 (33%) stage I tumours showed LOH at 5q, four of which also harboured *TP53* mutations suggestive of the involvement of these loci in early stages of the development of some ovarian cancers. There was an increase in the incidence of both 5q and *TP53* mutation with advancing stage, although this increase was not statistically significant. With respect to the

**Figure 1** Chromosome 5q allelic deletion pattern for ovarian tumours 47, 71, 86, 95 and 151 showing partial LOH of 5q. A full description of the LOH in these tumours is detailed in Table II. □, no LOH; ■, LOH. For each informative locus, the autoradiograph of the normal (N) and tumour (T) DNA PCR is shown to the right. Alleles for marker D5S424 and D5S644 were resolved on 8% non-denaturing polyacrylamide gels and D5S346 and MFD116 were resolved on 6% denaturing polyacrylamide gels.

main histological subtypes, 5q LOH is perhaps of less relevance in mucinous tumours since LOH was detected in only 20% (1/5) of the mucinous adenocarcinomas compared with 61% (16/26) of serous and undifferentiated adenocarcinomas and 55% (5/9) of endometrioid carcinomas. Among the other histological subtypes and borderline and benign tumours only one of the two mixed Müllelrian tumours showed 5q LOH.

### Discussion

Deletions on chromosome 5 which include the *APC* gene have been observed in a variety of malignancies other than just colorectal cancer and include oesophageal, gastric, pancreatic and lung carcinomas (Boynton *et al*., 1992; D'Aminco *et al*., 1992; Hori *et al*., 1992*a,b*; Hosoe *et al*., 1994). In ovarian cancers, chromosome 5q LOH has been reported by some groups to be an infrequent event (Ehlen and Dubeau, 1990; Sato *et al*., 1991; Yang-Feng *et al*., 1993) while others have shown frequent deletions (Cliby *et al*., 1993; Allan *et al*., 1994). These discrepancies are most likely due to differences in the number, location and type of polymorphic markers used in each study as well as the small size of the tumour collections. The most comprehensive of these studies used five markers on each chromosomal arm and detected LOH in 50% of the 27 tumours examined (Allan *et al*., 1994). The LOH was consistent with the loss of *APC*, but no mutations were detected by SSCP in any of the exons containing published mutations suggesting that another gene was the target of the deletions.

In the present study we analysed for chromosome 5 LOH using eight microsatellite markers to verify the high frequency of LOH reported by some and refine the location of the putative 5q TSG. Consistent with the frequencies reported by Cliby *et al*. (1993) and Allan *et al*. (1994) we detected LOH on 5q in 23 of 49 ovarian tumours. Thirteen of these tumours exhibited LOH on only part of 5q including two with interstitial deletions permitting the refinement of the candidate TSG locus to the 22 cM region, flanked by D5S424 and D5S644 at 5q 13.1–21. This region is proximal

**Table III** Comparison between LOH on chromosome 5q and *TP53* mutation[a]

| | TP53 *mutation*[a] | TP53 *normal* |
|---|---|---|
| 5q LOH | 18 | 5 |
| 5q Het | 4 | 22 |

[a]Correlation 0.631; *P*-value <0.001; 90% confidence interval (CI) (0.462–0.756). The correlations and their *P*-values were calculated by Spearman's rank correlation.

**Table II** Tumour clinical, chromosome 5 LOH and *TP53* mutation data

| *Tumour number* | *Type*[a] | *Stage* | *S417* | *S118* | *S424* | *S644* | *S346* | *IL9* | *S399* | *S209* | TP53 *mutation*[b] | *Codon, nucleotide and amino acid change*[c] |
|---|---|---|---|---|---|---|---|---|---|---|---|---|
| 11m | AC/UD | Ia | Het[d] | Het | | | **LOH** | **NI** | | **LOH** | exon 7 | NS |
| 12m | SPAC | Ia | Het | **NI** | | | **LOH** | **NI** | | **NI** | exon 5 | NS |
| 13m | SPAC | Ia | NI | Het | | | **LOH** | **NI** | | **NI** | exon 5 | NS |
| 17m | AC/UD | III | **NI** | **LOH** | | | **LOH** | **LOH** | | **LOH** | exon 5 | NS |
| 21m | SPAC | na | **NI** | **NI** | | | **LOH** | **NI** | | **NI** | exon 7 | NS |
| 22 | SPAC | III | Het | **LOH** | **LOH** | **LOH** | **LOH** | **NI** | **NI** | **NI** | exon 6 | 220, TAG>TGT, Tyr>Cys |
| 26 | SPAC | III | **NI** | **LOH** | **LOH** | **LOH** | **LOH** | **LOH** | **NI** | **LOH** | exon 7 | 242, TGC>TGG, Cys>Trp |
| 27 | AC/UD | I | Het | **LOH** | **NI** | **LOH** | **LOH** | **LOH** | **NI** | **NI** | n | |
| 30 | EC | III | **NI** | **NI** | **LOH** | **NI** | **NI** | **LOH** | **NI** | **NI** | exon 8 | 273, CGT>TGT, Arg>Cys |
| 32 | SPAC | III | Het | **NI** | **LOH** | **LOH** | **LOH** | **NI** | **NI** | **NI** | n | |
| 36 | EC | Ia | Het | **NI** | **LOH** | **LOH** | **LOH** | **LOH** | **LOH** | **LOH** | n | |
| 43 | AC/UD | II | **NI** | **LOH** | **NI** | **LOH** | **LOH** | **LOH** | **LOH** | **NI** | exon 5 | 157, GTC>GAC, Val>Asp |
| 45 | SPAC | III | **NI** | **NI** | **LOH** | **NI** | **LOH** | **LOH** | **NI** | **NI** | exon 6 | 196, GGA>TGA, Arg>Stop |
| 47 | AC/UD | II | | | **Het** | **LOH** | | | **NI** | | exon 5 | 179, CGC>CAC, Arg>His |
| 49 | MMT | III | Het | **LOH** | **LOH** | **LOH** | **LOH** | **NI** | **LOH** | **LOH** | exon 8 | 276, GCC>GC, frame shift |
| 63 | AC/UD | na | **LOH** | **NI** | **LOH** | **NI** | **LOH** | **LOH** | **LOH** | **LOH** | exon 7 | 242, TGC>GGC, Cys>Gly |
| 71 | SPAC | IIa | Het | **NI** | **LOH** | **LOH** | Het | NI | NI | Het | exon 7 | NS |
| 86 | SPAC | IIIa | Het | Het | **LOH** | **LOH** | Het | NI | Het | Het | exon 5 | 151, CCC>CGC, Pro>Arg |
| 95 | EC | II | | | Het | **LOH** | | | **NI** | | n | |
| 121 | MAC | III | | | **NI** | **LOH** | | | **NI** | | exon 5 | NS |
| 131 | SAC | III | | | **LOH** | **LOH** | | | **LOH** | | exon 8 | NS |
| 146 | EC | Ic | | | **LOH** | **NI** | | | **LOH** | | n | |
| 151 | EC | IIc | | | **LOH** | Het | | | NI | | exon 7 | NS |
| 2m | BSA | Ic | Het | NI | Het | Het | NI | NI | Het | | n | |
| 4m | SA | na | Het | NI | | | NI | Het | | NI | n | |
| 10m | EC | Ia | NI | NI | | | Het | Het | | Het | n | |
| 14 | SPAC | IIIb | Het | Het | NI | NI | Het | Het | Het | Het | n | |
| 15m | SPAC | IIb | Het | NI | | | Het | NI | | Het | n | |
| 16m | CCC | III | Het | NI | | | Het | NI | | Het | n | |
| 18m | SPAC | III | Het | Het | | | Het | | | | n | |
| 19 | SPAC | III | Het | NI | Het | Het | Het | NI | NI | NI | n | |
| 20 | BSA | IIIa | Het | Het | Het | Het | Het | Het | Het | Het | n | |
| 23 | SPAC | I | Het | NI | Het | NI | Het | NI | Het | Het | n | |
| 40 | MAC | II | Het | NI | Het | NI | Het | NI | Het | Het | n | |
| 48 | SPAC | III | | | NI | Het | | | Het | | n | |
| 50 | MAC | I | Het | NI | Het | Het | NI | NI | Het | Het | n | |
| 60 | GCT | Ia | | | Het | Het | | | NI | | n | |
| 70 | EC | Ia | Het | Het | Het | Het | Het | NI | | Het | n | |
| 75 | MA | na | | | NI | Het | | | | | n | |
| 80 | MAC | Ia | | | NI | Het | | | Het | | n | |
| 97 | MMT | III | | | Het | Het | | | Het | | exon 7 | NS |
| 114 | EC | Ia | | | Het | NI | | | NI | | n | |
| 119 | SPAC | Ic | | | Het | Het | | | Het | | n | |
| 122 | AC/UD | III | | | Het | NI | | | NI | | exon 5 | 174, AGG>AAG, Arg>Lys |
| 124 | GCT | III | | | NI | Het | | | Het | | n | |
| 128 | EC | Ic | | | Het | Het | | | NI | | n | |
| 134 | SPAC | III | | | Het | Het | | | Het | | exon 5 | 166, ins A, frame shift |
| 135 | SPAC | Ic | | | Het | Het | | | Het | | exon 5 | 156, 12 bp deletion |
| 144 | MAC | Ic | | | Het | Het | | | NI | | n | |

[a]AC/UD, adenocarcinoma, undifferentiated lineage; SPAC, serous papillary (cyst) adenocarcinoma (including serous carcinoma, papillary carcinoma and serous adenocarcinoma); MAC, mucinous adenocarcinoma; EC, endometrioid carcinoma; CCC, clear cell carcinoma; MMT, mixed Müllerian tumour; GCT, granulosa cell tumour; SPA, serous papillary adenoma; BSA, borderline serous adenoma. [b]*TP53* mutation in the exon indicated as determined by SSCP analysis. [c]Codon, nucleotide change and amino acid alteration is indicated. NS, not sequenced. [d]Het, constitutional heterozygosity without loss; LOH, constitutional heterozygosity with loss in the tumour DNA; NI, constitutional homozygosity and therefore uninformative with respect to allelic loss. Bold entries indicate the maximum extent of allelic deletion in each tumour.

Table IV Association between LOH on 5q and *TP53* mutation with tumour stage

| *Tumour stage* | TP53 *mutation*[a] | *5q LOH*[b] | TP53 *mutations/5q LOH*[c] |
|---|---|---|---|
| I | 4/18 (22%) | 6/18 (33%) | 3/6 (50%) |
| II | 4/7 (57%) | 5/7 (71%) | 4/5 (80%) |
| III | 12/20 (60%) | 10/20 (50%) | 9/10 (90%) |
| Unstaged | 2/4 (50%) | 2/4 (50%) | 2/2 (100%) |
| Totals[d] | 22/49 (45%) | 23/49 (47%) | 18/23 (78%) |

[a]Numbers of tumours with *TP53* mutation over the number of tumours of the stage indicated; figures in brackets are percentages. [b]Number of tumours with LOH anywhere on chromosome 5q divided by the total number of tumours of that stage with percentages in brackets. [c]Number of tumours with *TP53* mutation divided by the number of tumours with 5q LOH with percentages in brackets. [d]Number of tumours of all stages with the indicated property.

to *APC*, thereby excluding it as the candidate TSG, consistent with the absence of APC mutations in ovarian cancer reported by Allan *et al.* (1994).

LOH on 5q occurred in six (33%) stage I tumours, suggesting that it may be an early event in the development of certain ovarian cancers. This finding is inconsistent with the study by Allan *et al.* (1994), who concluded 5q LOH was a late event in ovarian carcinogenesis. However, their conclusion was based on the absence of LOH in only three low-grade tumours, highlighting a difficulty encountered in studies of this type in ovarian cancer in which low-grade and early-stage tumours are relatively uncommon. Nevertheless, such studies are vital if the sequence of molecular genetic events in ovarian tumorigenesis is to be unravelled.

Comparison of the presence of LOH on chromosome 5 with mutation in *TP53* revealed a significant association between the two genetic events ($P<0.001$). A similar observation has been reported in colorectal carcinomas (Smith *et al.*, 1995), but this is more likely to reflect an association with *APC* inactivation than with another 5q TSG. Although the association between 5q LOH and *TP53* mutation in ovarian cancer is striking, caution must be exercised in attributing this to a functional link between *TP53* and the putative 5q TSG as this might simply reflect generalised chromosomal instability in tumours with advancing stage. Only when the 5q TSG is cloned and it can be examined for specific inactivating mutations will it be possible to determine the true relationship between the two events.

**Acknowledgements**
We are grateful to Dr Ben Oostra for his help and advice on the LOH analysis, Mr David Hitchin for help with statistical analysis. This study was supported by grants from the Cancer Research Campaign, South Thames Regional Health Authorities and the Wessex Medical Trust.

### References

ALLAN GJ, COTTRELL S, TROWSDALE J AND FOULKES WD. (1994). Loss of heterozygosity on chromosome 5 in sporadic ovarian carcinoma is a late event and is not associated with mutations in APC at 5q21-22. *Hum. Mut.*, **3**, 283-291.

BOYNTON RF, BLOUNT PL, YIN J, BROWN VL, HUANG Y, TONG Y, MCDANIEL T, NEWKIRK C, RESAU JH, RASKIND WH, HAGGITT RC, REID B AND MELTZER SJ. (1992). Loss of heterozygosity involving the *APC* and *MCC* genetic loci occurs in the majority of human esophageal cancers. *Proc. Natl Acad. Sci. USA*, **89**, 3385-3388.

CAMPBELL IG, NICOLAI HM, FOULKES WD, STAMP GW, ALLAN G, BOYER CM, SENGER G, JONES K, BAST RC JR., SOLOMON E, TROWSDALE J AND BLACK DM. (1994). A novel gene encoding a B-box protein within the *BRCA1* region at 17q21.1. *Hum. Mol. Genetics*, **3**, 589-594.

CLIBY W, RITLAND S, HARTMANN L, DODSON M, HALLING KC, KEENY G, PODRATZ KC AND JENKINS RB. (1993). Human epithelial ovarian cancer alleloptype. *Cancer Res.*, **53**, 2393-2398.

D'AMINCO D, CARBONE DP, JOHNSON BE, MELZER SJ AND MINNA J. (1992). Polymorphic sites within the MCC and APC loci reveal very frequent loss of heterozygosity in human small lung cancer. *Cancer Res.*, **52**, 1996-1999.

EHLEN T AND DUBEAU L. (1990). Loss of heterozygosity on chromosome segments 3p, 6q and 11p in human ovarian carcinomas. *Oncogene*, **5**, 219-223.

ENGLEFIELD P, FOULKES WD AND CAMPBELL IG. (1994). Loss of heterozygosity on chromosome 22 in ovarian carcinoma is distal to and is not accompanied by mutations in NF2 at 22q12. *Br. J. Cancer*, **70**, 7486-7488.

FOULKES WD, RAGOUSSIS J, STAMP GWH, ALLAN GJ AND TROWSDALE J. (1993*a*). Frequent loss of heterozygosity on chromosome 6 in human ovarian carcinoma. *Br. J. Cancer*, **67**, 551-559.

FOULKES WD, BLACK DM, STAMP GWH, SOLOMON E AND TROWSDALE J. (1993*b*). Very frequent loss of heterozygosity throughout chromosome 17 in sporadic ovarian cancer. *Int. J. Cancer*, **54**, 220-225.

FOULKES WD, ENGLEFIELD P AND CAMPBELL IG. (1994). Mutation analysis of *RASK* and the 'FLR exon' of *NF1* in sporadic ovarian carcinoma. *Eur. J. Cancer*, **30A**, 528-530.

FOULKES WD, STAMP GWH, AFZAL S, LALANI N, MCFARLANE CP, TROWSDALE J AND CAMPBELL IG. (1995). MDM2 overexpression is rare in ovarian carcinoma irrespective of *TP53* mutation status. *Br. J. cancer*, **72**, 883-888.

GARDNER MJ AND ALTMAN DG. (1989). *Statistics with Confidence*. BMJ: London.

GYAPAY G, MORISSETTE J, VIGNAL A, DIB C, FIZAMES C, MILLASSEAU P, MARC S, BERNARDI G, LATHROP M AND WEISSENBACH J. (1994). The 1993-94 Genethon human genetic linkage map. *Nature Genet.*, **7**, 246-249

HORII A, NAKATSURU S, MIYOSHI Y, ICHII S, NAGASE H, KATO Y, YANAGISAWA A AND NAKAMURA Y. (1992*a*). The *APC* gene, responsible for familial adenomatous polyposis, is mutated in human gastric cancer. *Cancer Res.*, **52**, 3231-3233.

HORII A, NAKATSURU S, MIYOSHI Y, ICHII S, NAGASE H, ANDO H, YANAGISAWA A, TSUCHIYA E, KATO Y AND NAKAMURA Y. (1992*b*). Frequent somatic mutations of APC gene in human pancreatic cancer. *Cancer Res.*, **52**, 6696-6698.

HOSOE S, UENO K, SHIGEDO Y, TACHIBANA I, OSAKI T, KUMAGAI T, TANIO Y, KAWASE I, NAKAMURA Y AND KISHIMOTO T. (1994). A frequent deletion of chromosome 5q21 in advanced small cell and non-small cell carcinoma of the lung. *Cancer Res.*, **54**, 1787-1790.

KOHLER MF, KERNS B-JM, HUMPHREY PA, MARKS JR, BAST RC AND BERCHUCK A. (1993*a*). Mutation and overexpression of p53 in early-stage epithelial ovarian cancer. *Obstet. Gynecol.*, **81**, 643-650.

KOHLER MF, MARKS JR, WISEMAN RW, JACOBS IJ, DAVIDOFF AM, CLARKE-PEARSON DL, SOPER JT, BAST RC AND BERCHUCK A. (1993*b*). Spectrum of mutation and frequency of allelic deletion of the p53 gene in ovarian cancer. *J. Natl Cancer Inst.*, **85**, 1513-1519.

MILNER BJ, ALLAN LA, ECCLES DM, KITCHENER HC, LEONARD RCF, KELLY KF, PARKIN E AND HAITES NE. (1993). p53 is a common genetic event in ovarian carcinoma. *Cancer Res.*, **53**, 2128-2132.

OKAMOTO A, SAMESHIMA Y, YOKOYAMA S, TERASHIMA Y, SUGIMURA T AND TERADA M. (1991). Frequent allelic losses and mutations of the p53 gene in human ovarian cancer. *Cancer Res.*, **51**, 5171-5176.

SATO T, SAITO H, MORITA R, KOI S AND NAKAMURA Y. (1991). Allelotype of human ovarian cancer. *Cancer Res.*, **51,** 5118–5122.
SHEPHERD JH. (1989). Revised FIGO staging for gynaecological cancer. *Br. J. Obst. Gynaecol.*, **96,** 889–892.
SMITH DR, KHINE K, CHAN CS AND GOH HS. (1995). Tumour suppressor genes in colorectal carcinomas: p53 inactivation is highly associated with allelic loss of chromosome 5q. *Int. Oncol.*, **5,** 539–546.
TAVASSOLI M, RUHRBERG C, BEAUMONT V, REYNOLDS K, KIRKHAM N, COLLINS WP AND FARZANEH F. (1993). Whole chromosome 17 loss in ovarian cancer. *Genes, Chrom. Cancer*, **8,** 195–198.
WEINBERG R. (1992). Tumour suppressor genes. *Science*, **254,** 1138–1145.
YANG-FENG TL, HAN H, CHEN KC, LI SB, CLAUS EB, CARCAUGIN ML, CHAMBERS SK, CHAMBERS JT AND SCHWARTZ PE. (1993). Allelic loss in ovarian cancer. *Int. J. Cancer*, **54,** 546–551.